# Efficient Removal of PFASs Using Photocatalysis, Membrane Separation and Photocatalytic Membrane Reactors

**DOI:** 10.3390/membranes14100217

**Published:** 2024-10-14

**Authors:** Nonhle Siphelele Neliswa Mabaso, Charmaine Sesethu Tshangana, Adolph Anga Muleja

**Affiliations:** Institute for Nanotechnology and Water Sustainability, College of Science, Engineering and Technology, University of South Africa, Johannesburg 1709, South Africa

**Keywords:** perfluorooctane sulfonate (PFOS), perfluorooctanoic acid (PFOA), electrostatic interactions, granular activated carbon, nanofiltration, reverse osmosis, PFAS destructive techniques

## Abstract

Perfluoroalkyl and polyfluoroalkyl substances (PFASs) are persistent compounds characterized by stable C−F bonds giving them high thermal and chemical stability. Numerous studies have highlighted the presence of PFASs in the environment, surface waters and animals and humans. Exposure to these chemicals has been found to cause various health effects and has necessitated the need to develop methods to remove them from the environment. To date, the use of photocatalytic degradation and membrane separation to remove PFASs from water has been widely studied; however, these methods have drawbacks hindering them from being applied at full scale, including the recovery of the photocatalyst, uneven light distribution and membrane fouling. Therefore, to overcome some of these challenges, there has been research involving the coupling of photocatalysis and membrane separation to form photocatalytic membrane reactors which facilitate in the recovery of the photocatalyst, ensuring even light distribution and mitigating fouling. This review not only highlights recent advancements in the removal of PFASs using photocatalysis and membrane separation but also provides comprehensive information on the integration of photocatalysis and membrane separation to form photocatalytic membrane reactors. It emphasizes the performance of immobilized and slurry systems in PFAS removal while also addressing the associated challenges and offering recommendations for improvement. Factors influencing the performance of these methods will be comprehensively discussed, as well as the nanomaterials used for each technology. Additionally, knowledge gaps regarding the removal of PFASs using integrated photocatalytic membrane systems will be addressed, along with a comprehensive discussion on how these technologies can be applied in real-world applications.

## 1. Introduction

Over the last few years, considerable research has been geared towards the removal of perfluoroalkyl and polyfluoroalkyl substances (PFASs) from the environment. To this end, various methods have been investigated in the degradation and removal of PFASs from the environment using destructive or non-destructive techniques [1,2]. Non-destructive techniques, also known as sorption techniques, do not degrade PFASss but separate them from various matrices. These techniques include ion exchange (IE) and adsorption using granular activated carbon (GAC) which have demonstrated effective performance in removing PFASs from real water sources. In Sweden, AE resins and GAC removed an average of 66% and 62% of multiple long- and short-chain PFASs from municipal drinking water, respectively [3]. In another study, bamboo-derived activated carbon (BAC) and the AE resin IRA67 removed >90% of perfluorohexanoic acid (PFHxA), perfluoroheptanoic acid (PFHpA) and perfluorooctanoic acid (PFOA) from wastewater [4]. These methods have drawbacks related to additional costs associated with the need to regenerate the sorbents used, which often requires highly toxic organic solvents [3,4].

Membrane separation is used to remove PFASs from water. Nanofiltration (NF) and reverse osmosis (RO) membranes are often used, owing to their good removal efficiency and effectiveness in removing both long- and short-chain PFASs. However, like the other sorption techniques, membranes also do not degrade PFASs but instead produce concentrated streams of PFASs [5,6,7,8,9]. Additionally, membranes are susceptible to fouling, which results in the need for backwashing, an increase in energy consumption due to the use of high pressures and the reduced lifespan of membranes [7].

To mitigate the limitations associated with membranes, alternative techniques whose emphasis is on the degradation of PFASs are considered. These techniques include AOPs, plasma and electrochemical oxidation. Recently, photocatalysis has been an area of interest regarding the degradation of PFASs due to the ambient reaction conditions, minimal use for additional chemical additives and the recyclability of photocatalysts [10]. Although photocatalysis is promising, scaling up and the recovery of the photocatalyst remain two of the most significant drawbacks. This has prompted investigation into the use of hybrid systems combining photocatalysis and membrane separation to overcome these challenges [11]. Hybrid systems such as photocatalytic membrane reactors (PMRs) offer several advantages including reduced fouling, facilitating in the recovery of the photocatalyst. Additionally, PMRs facilitate the pre-concentration of PFASs, thus enabling effective degradation in a compact space; improve mass transfer between the photocatalyst and PFASs; and allow for continuous operation [12,13].

The review will examine factors influencing the photocatalytic degradation of PFASs. The performance of metal oxide photocatalysts employed in this process will be evaluated. The rejection of PFASs using membrane filtration will be discussed, including factors that affect membrane performance and the types of membranes utilized. Finally, the integration of these two techniques into hybrid systems for the development of photocatalytic membrane reactors will be explored.

## 2. Degradation of Per- and Polyfluoroalkyl Substances via Photocatalysis

Heterogeneous photocatalysis involves the use of various light sources and semiconductor materials to degrade persistent contaminants to mineralize them into innocuous substances. The photocatalytic process involves irradiating light of a specific wavelength onto the photocatalyst’s surface, causing electrons to be excited and thus migrate to the conduction band from the valence band. This results in the formation of charged electrons in the conduction band (e^−^) and holes (h^+^) in the valence band [14,15]. These charged carriers are then responsible for initiating the photocatalytic degradation process as they generate reactive oxygen species (ROS) including superoxide radicals (^•^O_2_^−^) or hydroperoxyl radicals (HO_2_^•^) and hydroxyl radicals (^•^OH), which together with the charge carriers react with the pollutant (PFASs in this case) to degrade them into mineralization products or shorter-chain by-products [15]. 

ROS are generally effective in degrading contaminants such as dyes and organic compounds [16,17,18]; however, they are not as effective in degrading PFASs due to the strong C-F bond in PFASs. The ^•^O_2_^−^ (E° = 1.83 V), ^•^OH (E° = 2.8 V) and hydroperoxyl (E° = 1.44 V) radicals are unable to break the C-F bond in PFASs directly because of their high bond energy of 107–118 kcal/mol [18,19,20]. Therefore, the degradation of PFASs using photocatalysis (Equations (1)–(4)) typically begins when the photogenerated holes (h^+^) on the photocatalyst’s surface accept electrons from the absorbed C_7_F_15_COO^−^ generating PFAS radicals (Equation (1)) [21,22]. These radicals then undergo decarboxylation to form C_7_F_15_^•^ radicals (Equation (2)), which rapidly react with ^•^OH radicals (which were formed on the surface of the photocatalyst after H_2_O molecules adsorbed on the photocatalyst’s surface react with the h^+^ formed in the valence band) to produce thermodynamically unstable alcohols (Equation (3)). These alcohols undergo hydrolysis and HF elimination (Equations (3) and (4)) [22]. This process repeats until the PFAS molecules are mineralized to CO_2_ and fluoride ions. Several factors can influence the degradation and defluorination rate of PFASs, and below, we interrogate those factors.

C_7_F_15_COO^−^ + h^+^ ⟶ C_7_F_15_COO^•^ (1)

C_7_F_15_COO^•^ ⟶ C_7_F_15_^•^ + CO_2_ (2)

C_7_F_15_^•^ + ^•^OH/H_2_O ⟶ C_6_F_13_COH (3)

C_6_F_13_COH ⟶ C_6_F_13_COF + HF (4)

### 2.1. Factors Affecting the Degradation and Defluorination of Per- and Polyfluoroalkyl Substances

#### 2.1.1. Light Source

**Figure 1:**
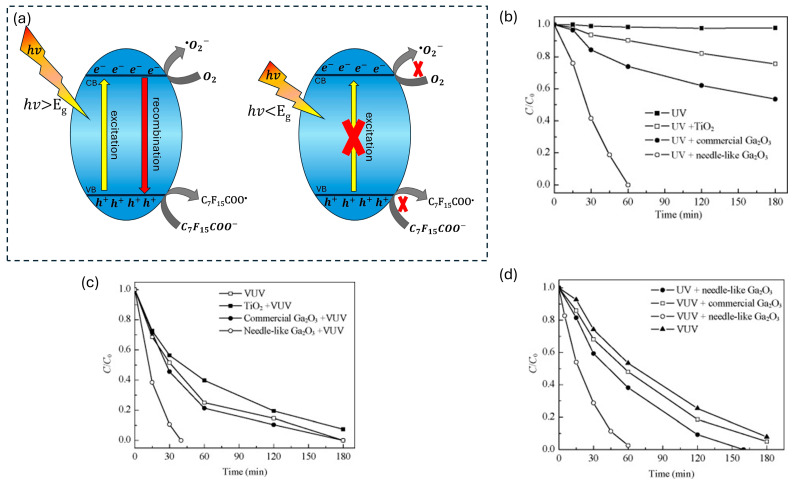
(**a**) Schematic representation of the efffect of light on photocatalytic degradation of PFOA (this work), (**b**) degradation of PFOA in pure water using UV irradiation, (**c**) pure water using VUV irradiation and (**d**) sewage water using UV and VUV irradiation (**b**–**d** reproduced with permission from Ref. [24]).

The light source used in the photocatalytic degradation of PFASs plays a crucial role in the rate of degradation by influencing the production of charge carriers responsible for breaking down PFASs. When the photocatalyst absorbs light, electrons are excited from the valence band to the conduction band, generating electron–hole pairs necessary for the degradation process [23]. For this excitation to occur, sufficient energy from the light source is essential to drive this process. The photon energy (hv) must be greater than or equal to the band gap energy (Eg) of the photocatalyst for the excitation to occur. As shown in Figure 1a, if hv<Eg, the photons do not have enough energy to excite electrons, and no photocatalytic activityoccurs. However, when hv>Eg, the photocatalyst is activated, producing electron–hole pairs that generate reactive species for degrading PFASs.

In an experiment where different light sources were used in the degradation of PFOA, vacuum UV (VUV) light (*λ* = 185 nm) demonstrated greater effectiveness in degrading PFOA compared to UV light (*λ* = 254 nm). As illustrated in Figure 1b, UV light resulted in a negligible degradation of PFOA. On the other hand, VUV light achieved nearly 90% degradation within 180 min (Figure 1c) without the need for a photocatalyst, highlighting VUV’s efficiency in breaking down PFOA. The performance of VUV was also tested in real water samples, where it maintained its effectiveness, indicating that organic matter in wastewater did not impact its degradation ability (Figure 1d) [24].

This phenomenon can be explained by Equation (5) which shows the inverse relationship between photon energy and wavelength. Typically, light with smaller wavelengths tends to have more energy than those with a longer wavelength.

(5) $$hv=\frac{hc}{\lambda }$$

whereby hv is the photon energy, h is Planck’s constant, c is the speed of light and λ is the wavelength of light.

Elsewhere, Xu et al. [25] conducted a study where they observed that the degradation of PFOA took 120 min under UV light (254 nm) and took 60 min under 185 nm VUV light. From this, it was concluded that VUV is a more effective light source than UV in degrading PFASs as it has higher energy to directly photolyze PFASs. It is worth noting that the energy consumption needed to degrade PFOA in synthetic water using needle-like Ga_2_O_3_/VUV was determined to be 94.6 kJ/μmol which was much less than that when using other methods such as sonochemical degradation which had a consumption of 1300 kJ/μmol [26]. The energy consumption of Ga_2_O_3_/VUV in degrading PFOA in wastewater was also found to be 108.6 kJ/μmol which was much lower than that of Cheng et al. [27] who investigated the performance of sonolysis in degrading PFOA in groundwater, where it was confirmed that the energy consumption was 4099 kJ/μmol.

The use of visible light in degrading PFASs is limited by large band gaps of photocatalysts that are mostly used. However, a group of bismuth-based photocatalysts (BiOX) have emerged as promising photocatalysts for the degradation of perfluorooctanoic acid (PFOA) due to their unique layered structure and excellent visible light response. These materials exhibit a suitable band gap, typically in the range of 1.8 to 2.8 eV, which allows them to efficiently utilize visible light for photocatalytic processes [28]. Studies have shown that BiOX photocatalysts can achieve a significant degradation of PFOA under visible light, offering a cost-effective approach to mitigate PFAS pollution in water [28,29,30].

#### 2.1.2. pH

**Figure 2:**
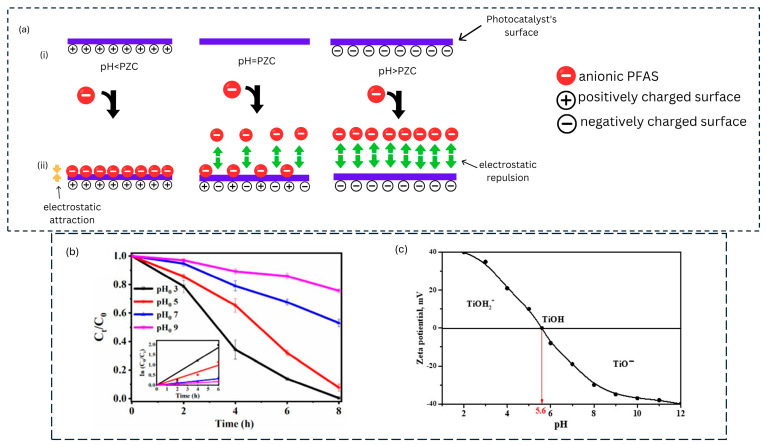
Schematic representation of the (**a**) Effect of pH on photocatalyst’s surface charge (i) and interaction of anionic PFASs and photocatalyst’s surface at different pH values (ii) (this work). (**b**) Effect of pH on photocatalytic degradation of PFOA and (**c**) zeta potential of TiO_2_ at various pH values (**b** and **c** reproduced with permission from Ref. [35]).

The pH of a solution impacts the rate of degradation of PFASs as it influences (i) the dissociation of PFAS molecules, (ii) the surface chemistry of the photocatalyst and (iii) the interaction between the photocatalyst and PFAS molecules. At lower pH values, most photocatalysts such as TiO_2_ are positively charged (pH < pzc) on the surface, while PFAS molecules such as PFOA and PFOS exist in their anionic form due to the carboxylate and sulfonate groups (Figure 2a(i)) [31]. Electrostatic interactions encourage PFASs to be adsorbed on the surface of the photocatalyst where the degradation occurs (Figure 2a(ii)). In alkaline conditions (pH > pzc), PFOA is still anionic, and the photocatalyst is also negatively charged, which leads to repulsive forces, thus limiting adsorption, which in turn hinders degradation efficiency [32,33]. 

Various studies have demonstrated a trend where there is an inverse behaviour in the photocatalytic degradation of PFOA with increasing pH wherein an increase in the pH of the solution results in a decrease in photocatalytic degradation [32,33,34,35]. For instance, Xu et al. [35] assessed the degradation of PFOA using TiO_2_ and peroxymonosulfate (PMS) at various pH (3, 5, 7 and 9) values under visible light. The highest degradation was observed at pH 3 where 100% of PFOA was degraded in 8 h (Figure 2b). The degradation decreased to 90% and 45% when the pH increased to 5 and 7 and further dropped to 24% at pH 9. This was attributed to the formation of hydroxyl radicals at alkaline conditions which are not strong enough to break up the C-F bond of PFOA. Secondly, the point of zero charge of TiO_2_ was determined to be 5.6, meaning that the surface of TiO_2_ was positively charged when the solution pH was less than 5.6, resulting in an attraction between the photocatalyst’s surface and the PFOA molecules, as observed in (Figure 2c). 

#### 2.1.3. Concentration

**Figure 3:**
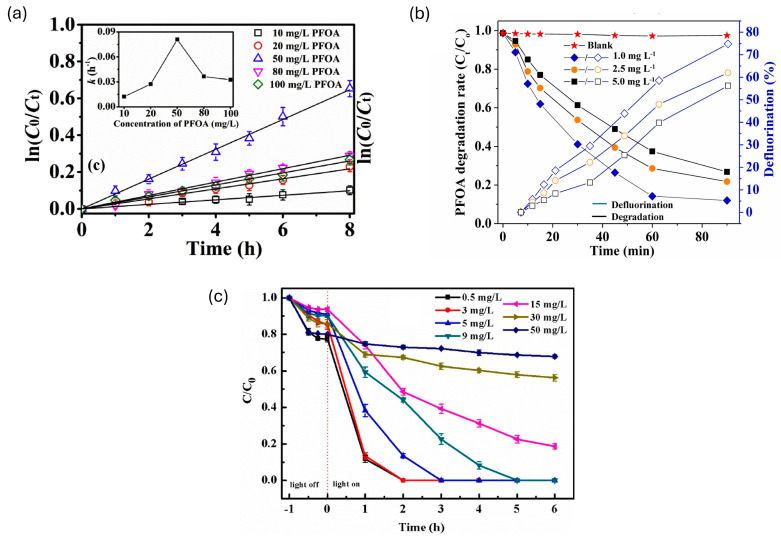
The effect of solute concentration on the degradation efficiency of PFOA with (**a**) Pb-BFO/0.5%rGO (reproduced with permission from Ref. [32]. (**b**) duo functional tri-metallic-oxide (f-TMO) photocatalyst (reproduced with permission from Ref. [37]) and (**c**) BiOI@Bi_5_O_7_I heterojunction photocatalyst (reproduced with permission from Ref. [38]).

The concentration of PFASs in solution greatly influences their photocatalytic degradation, owing to the adsorption on the surface of the photocatalyst. At lower concentrations, there are fewer molecules available to be adsorbed on the photocatalyst’s surface which will in turn decrease the degradation rate of PFASs since the reaction transpires at the surface [32,36]. An inverse relationship between the initial concentration of PFASs and their photocatalytic degradation has been found in which they decrease with an increasing concentration of PFOA [36]; this trend was also observed across various studies (Figure 3) [32,37,38].

For instance, Shang et al. [32] studied the degradation of PFOA using a bismuth ferrite (BFO) photocatalyst doped with lead (Pb) and reduced graphene oxide (rGO) (Pb-BFO/0.5%rGO). The degradation of PFOA was found to increase when the concentration of PFOA was increased from 10 mg/L to 50 mg/L; this was observed with an increase from the rate constants’ *k* values from 0.012/h to 0.081/h. However, when the concentration of PFOA was further increased to 100 mg/L, the *k* value reduced to 0.033/h, and this was caused by the oversaturation of the active sites available on the photocatalyst’s surface, resulting in less photons reaching the surface of the photocatalyst. Additionally, an increase in the concentration hinders photons from reaching the photocatalyst due to the adsorption of light by PFOA molecules.

#### 2.1.4. Photocatalyst Dosage

**Figure 4:**
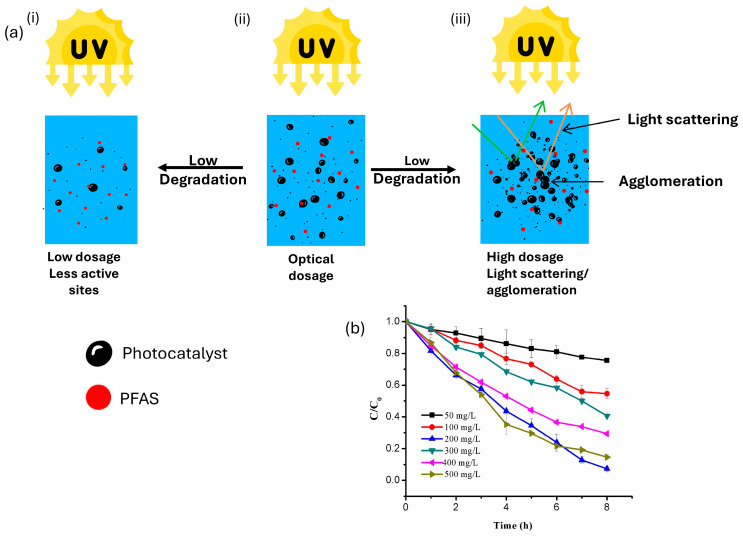
Schematic representation (**a**) of the degradation efficiency of PFASs at low photocatalyst dosage(i), optimal dosage(ii) and high dosage (iii) (this work). (**b**) Degradation of PFOA at various photocatalyst dosages (reproduced with permission from Ref. [41]).

Usually, an increase in the concentration of the photocatalyst increases the photocatalytic degradation of PFASss. Figure 4 depicts how increasing the photocatalyst dosage influences the degradation of PFOA, where the degradation initially increases with increasing dosage; however, it reaches an optimal point, and then a decrease is observed. This can be explained by a few factors: initially, an increase in the photocatalyst dosage increases the active sites available for PFASs to adsorb, resulting in an elevated degradation efficiency (Figure 4a(i)) [32,39]. There is, however, an optimal photocatalyst dosage (Figure 4a(ii)), wherein a continual increase in dosage may not have much of an effect on degradation efficiency. This is due to the agglomeration of the catalyst occurring when there is excessive catalyst loading which in turn lowers the number of active sites available for the reaction to occur in [40].

Overdosage can also turn the solution turbid and lead to light scattering which will limit the activation of the photocatalyst, as seen in Figure 4a(iii) [40]. This trend was observed across various photocatalysts and studies (Figure 4b) [32,38,39,40,41]. For example, Wang et al. [38] observed a similar trend where the degradation of PFOA was reported using BiOI@Bi_5_O_7_I as the photocatalyst whereby the photocatalyst dosage was varied between 0.1 and 2.0 g/L. The degradation efficiency was found to increase from 30% to 82% when the dosage increased from 0.1 g/L to 0.7 g/L; however, this decreased to 50% when the dosage was further increased to 2.0 g/L due to the aggregation of the photocatalyst and light scattering effects, as seen in [39].

### 2.2. Nanoparticles Used for Photocatalytic Degradation of PFASs

#### 2.2.1. Metal Oxides

Numerous metal oxides have been investigated for the photocatalytic degradation of PFASs with TiO_2_ being the most investigated photocatalyst. However, the performance of TiO_2_ has shown low efficiency in degrading PFOA under harsh or mild conditions, and thus, other metal oxides have been sought including In_2_O_3_ [42], Ga_2_O_3_ [43], CeO_2_ [44] and ZnO [4] which are summarized in Table 1.

The photocatalytic performance of the photocatalysts is significantly affected by either their atomic structure or morphology, which affects coordination, specific surface area and surface atomic arrangements. As previously described, the photocatalytic degradation of PFASs occurs on the surface of the photocatalyst; therefore, a high surface area is advantageous for the activity of the photocatalyst [46]. For example, the photocatalytic degradation of PFOA via photocatalysis requires the electrons from PFOA to be transferred to the photocatalyst, and thus, the morphology of the photocatalyst is postulated to play a crucial role in enhancing the rate of degradation [46]. 

**Figure 5:**
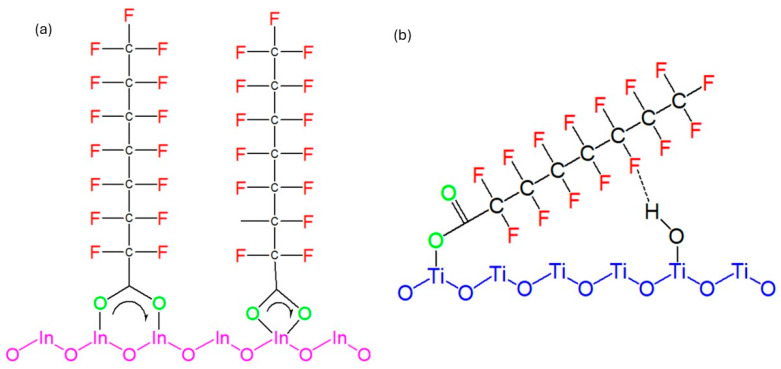
Configuration showing adsorption of PFOA on (**a**) In_2_O_3_ and (**b**) TiO_2_.

Regarding In_2_O_3_, the chemical states on the surface indium and oxygen elements could have a significant impact on the adsorption of PFOA and subsequently its degradation. Given that PFOA molecules possess a carboxyl group on the end of their tail, PFOA molecules add an oxygen atom from their carboxyl group into an oxygen vacant site on the surface of In_2_O_3_ to establish a close, tight bond with In_2_O_3_, as illustrated in Figure 5a; this is useful for the direct transfer of charge and successive photocatalytic degradation irradiation with UV.

With TiO_2_, the bonding between the terminal carboxyl group of PFOA and the photocatalyst occurs in a monodentate way, which then results in an arrangement of PFOA which is tilted onto the surface of TiO_2_. This results in the CF_2_ groups in the PFOA tail experiencing hydrogen bonding with the OH groups on the TiO_2_ surface, as shown in Figure 5b. The PFOA chains on the In_2_O_3_ surface are arranged in an ordered and tight configuration which promotes direct hole oxidation over water oxidation to hydroxyl radicals, leading to more efficient PFOA degradation on the irradiated surface of In_2_O_3_ than on TiO_2_ [47]. 

These results substantiated work conducted by Li et al. [46] who found that In_2_O_3_ materials (particularly porous microspheres) consist of a high density of oxygen vacant defects, results in an effective photocatalytic degradation of PFOA where the rate constants of In_2_O_3_ nanocubes, nanoplates, microspheres and TiO_2_ were 1.83/h, 4.45/h, 7.94/h and 0.106/h, respectively. In the study, the half-life of PFOA with microspheres of In_2_O_3_ was determined to be 5.3 min, while that of TiO_2_ was 391 min [46]. 

Like In_2_O_3_, Ga_2_O_3_ also binds in a bidentate configuration with PFOA using the terminal carboxyl group aiding PFOA to be degraded directly by the photogenerated holes of Ga_2_O_3_ under UV irradiation. This was demonstrated by Shao et al. [43], where PFOA was degraded in water using sheaf-like Ga_2_O_3_ that completely degraded PFOA in 45 min. The rate constant was 4.85/h which was 44 times higher than that of TiO_2_ which had a rate constant of 0.11/h. 

#### 2.2.2. Modification of TiO_2_ to Improve Degradation of PFOA

TiO_2_ has low efficiency in degrading PFOA due to the fast recombination of charge carriers and a wide band gap (3.2 eV). However, its availability, low toxicity and low cost make it easier to scale up [48]. As a result, research strides have been made in modifying TiO_2_ to improve its efficiency in degrading PFASs either by doping with noble metals [49], transition metals [34] or carbon allotropes (i.e., reduced graphene oxide [50] or coating with multiwall carbon nanotubes (MCWNTs)) [51] (Table 2).

**Figure 6:**
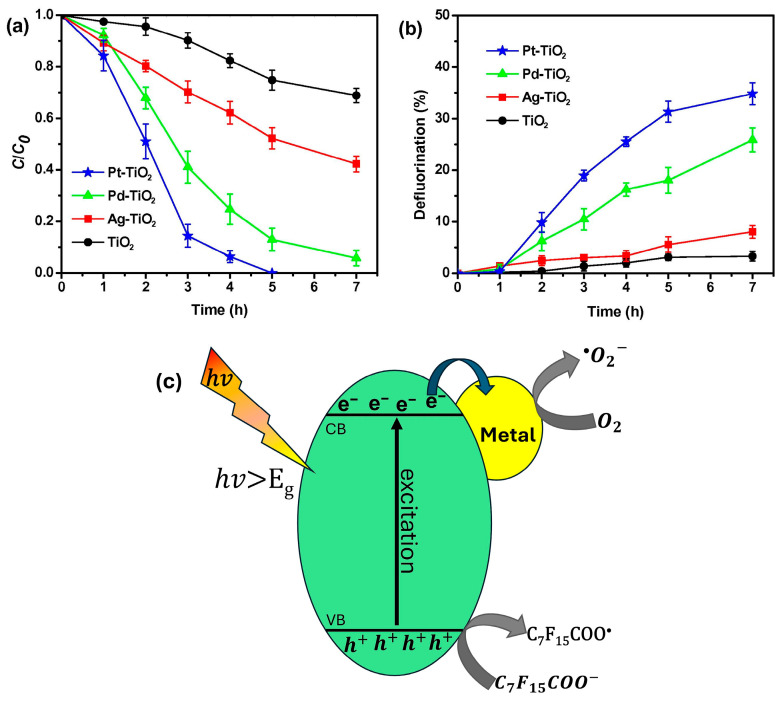
(**a**) Degradation and (**b**) defluorination of PFOA using noble metal-doped TiO_2_ ((**a**,**b**) reproduced with permission from Ref. [49]); (**c**) schematic representation of the electron trapping in metal-doped photocatalyst (this work).

Doping TiO_2_ with noble metals (M) improved its performance in degrading PFOA (Figure 6a,b). The noble metals were found to delay the recombination of the charge carriers in TiO_2_ as they acted as trapping sites which captured and held the excess electrons, thus allowing the holes present in the valence band to degrade PFOA, as illustrated in Figure 6c [49]. Li et al. [49] demonstrated that doping TiO_2_ with Platinum (Pt-TiO_2_), Palladium (Pd- TiO_2_) and silver (Ag-TiO_2_) resulted in rate constants that were 12.5, 7.5 and 2.2 times higher than that of TiO_2_ alone, respectively. Transition metal doping also improved the performance of TiO_2_ which, like the noble doped metals, iron (Fe-TiO_2_) and Copper (Cu-TiO_2_) systems produced electron traps to capture excess electrons by reducing the recombination of charge carriers during photocatalytic reactions and consequently enhancing the degradation of PFOA [34].

TiO_2_ supported on various carbon materials like multiwalled carbon nanotubes (MWCNTs), graphene and rGO generally demonstrated an improved direct interaction with PFOA during photocatalysis. This enhancement was ascribed to the carbon materials having high adsorption capacity which was facilitated by their large surface area and surface functional groups. Additionally, carbon materials can capture photogenerated electrons from TiO_2_, leading to a reduction in the recombination process of electrons and holes during photocatalysis. TiO_2_-rGO and TiO_2_-GO degraded 99.2% and 93.6% of PFOA in 8 h, respectively. Meanwhile, TiO_2_ only degraded 16% when irradiated with UV light. The specific surface areas for TiO_2_-rGO and TiO_2_-GO were found to be 121.3 m^2^/g and 115.5 m^2^/g, which was two times larger than that of TiO_2_ which was 50 m^2^/g [41].

#### 2.2.3. Adsorptive Photocatalysts

Adsorptive photocatalysts are based on the “concentrate and destroy” concept wherein the photocatalyst adsorbs PFASs, which is often the pre-concentration step [18]. Subsequently, when irradiated with light, degradation occurs. This also allows for the regeneration of the photocatalyst without using chemical regeneration, which reduces the high costs associated with implementing this technology. Furthermore, this can provide a solution to overcoming the drawback of applying photocatalysis at full scale, which is energy-extensive due to having to subject large volumes of water to irradiation but also needing large equipment and volume reactors [52].

Applying photocatalysis in degrading PFASs in raw water is not practical due to the low concentrations (ng/L or μg/L levels) of PFASs detected in these waters. As such, adsorptive photocatalysts can be advantageous in PFAS applications as this will pre-concentrate PFASs from a large body of water onto a small volume of a photocatalyst. The photocatalyst will be irradiated with light, allowing for the degradation of the PFAS molecules. Considerable energy savings can be obtained by limiting the area that needs to be irradiated with light to just the photocatalyst surfaces, as opposed to treating large volumes of water, thus lowering energy-related operating. To achieve reliable results in a range of water treatment situations, the photocatalyst materials used in this approach must be optimized for stability and effectiveness. Therefore, the concept of using photocatalysts for PFAS degradation offers a promising pathway towards sustainable and energy-efficient water treatment solutions.

Zhu et al. [53] evaluated the degradation of Gen X using a Bi/TNTs@AC photocatalyst. The photocatalyst was synthesized by depositing an amount of bismuth (Bi) on activated carbon (AC) supported by titanate nanotubes (TNTs). The collective interactions between Gen X and 3%Bi/TNTs@AC (i.e., hydrophobic, anion–π interactions and Lewis acid–base) promoted the adsorption of the (−COO^−^) group of Gen X onto the photocatalyst. The 3%Bi/TNT@AC photocatalyst adsorbed > 90% in the first 10 min and >99% within 60 min. Using Isotherm studies, it was determined that lower concentrations of Gen X led to higher adsorption levels due to the enhanced affinity of the photocatalyst to Gen X, and increasing the concentration lowers the selectivity of the photocatalyst. Based on this, it is apparent that the photocatalyst could be ideal in bulk water applications where the concentration of PFASs is at trace concentrations. The 3%Bi/TNTs@AC photocatalyst degraded >77.2% of Gen X with defluorination being 46.1% in 8 h under UV irradiation. Bi was also believed to act as a recombination centre effectively slowing down the recombination rate which then enhanced photocatalytic activity by facilitating the extended interaction between the charge carriers and Gen X.

Elsewhere, Li et al. [18] observed the degradation of PFOA using the adsorptive photocatalyst Fe/TNTs@AC. Adsorption kinetic studies were performed in the absence of light; over 95% of PFOA was adsorbed within 5 min, and this increased to over 99% within 60 min. This rapid and extensive adsorption was attributed to the synergistic effects of various adsorption mechanisms, including hydrophobic interactions and electrostatic interactions provided by AC, TNT and Fe. The adsorptive photocatalyst pre-concentrated PFOA onto the photocatalyst before it was exposed to UV light; this reduced the energy consumption which would be experienced when directly treating bulk water. After the pre-concentration of PFOA on the photocatalyst, it was exposed to UV irradiation, and 91.3% of PFOA was degraded in 4 h, and 62% was converted to F^−^ ions (defluorination).

## 3. Membrane Separation

**Figure 7:**
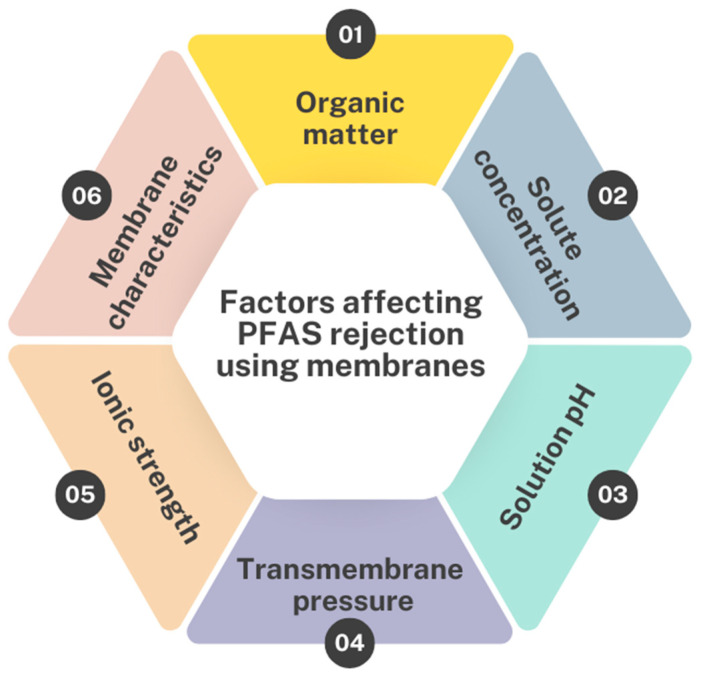
Factors affecting the removal of PFASs via membrane separation.

Membrane separation technologies offer promising solutions for the removal of PFASs from contaminated water sources owing to their high selectivity, efficiency and scalability [54,55,56]. These technologies depend on semi-permeable membranes to selectively separate pollutants based on their size, charge and hydrophobicity, thereby providing a sustainable approach to water purification. Several factors affect the efficiency of the separation process, which are summarized in Figure 7 and will be discussed in greater detail in the subsequent sections.

### 3.1. Factors Affecting Membrane Separation of Per- and Polyfluoroalkyl Substances

#### 3.1.1. Organic Matter

**Figure 8:**
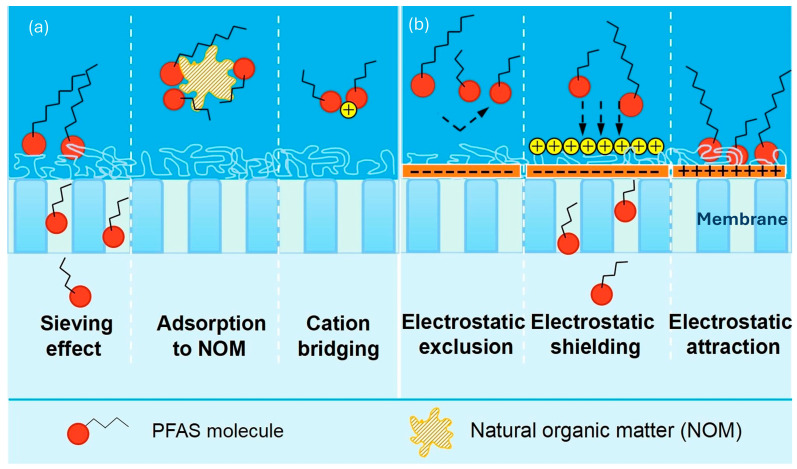
Schematic representation of PFAS rejection efficiency in the presence of organic matter, illustrating (**a**) size exclusion and (**b**) electrostatic interactions. Black arrows indicate electrostatic exclusion between PFAS molecules and the membrane surface, as well as electrostatic shielding caused by the adsorption of cations in solution, which shields the membrane surface. (Reproduced with permission from Ref. [58]).

The organic matter present in natural waters may influence the rejection of PFASs as well as compete with adsorption sites available in the membrane. This can also lead to membrane fouling which in turn will reduce the permeate flux. However, the rejection efficiency of the membrane has been found to increase especially in long-chain PFASs due to the organic matter binding to PFASs, increasing size exclusion. For example, Ma et al. [57] observed that the rejections of PFOA and PFBA increased with increasing humic acid (HA) and fluvic acid (FA) concentrations in feed solution due to the binding of HA and FA to PFASs, which resulted in the formation of larger complexes. However, the introduction of Bovine Serum Albumin (BSA) into the feed decreased the rejection of PFBA, while the rejection of PFOA increased. This difference was attributed to the distinct rejection mechanisms for each compound; PFOA was primarily rejected through size exclusion and PFBA through electrostatic repulsion which was reduced due to the interaction between the membrane surface and BSA, as shown in Figure 8a,b [58].

In the same vein, Zhao et al. [59] observed that the rejection of PFOS increased slightly from 94.1% to 95.1% as the concentration of HA in the feed solution was raised from 5 mg/L to 20 mg/L. It is important to note that this marginal increase in PFOS rejection suggested that size exclusion was already the predominant mechanism responsible for rejecting PFOS. However, the slight improvement in rejection efficiency was attributed to enhanced electrostatic exclusion. It was observed that the zeta potential of the membrane surface decreased with an increasing concentration of HA, thus indicating a stronger electrostatic repulsion between the negatively charged membrane and PFOS molecules, which are also negatively charged.

Wang et al. [60] noted an increase in the rejection of perfluorobutane sulfonate (PFBS) and PFOS in the presence of Sodium alginate (SA) or BSA. In the absence of BSA and SA, the rejection of PFBS and PFOS was found to be 48.9% and 89.6%, respectively; however, in the presence of SA or BSA, the rejection of PFOS improved to 91.4% and 95.6%, and for PFBS, they decreased to 46.3% and 68.6%, respectively. The observed effect was attributed to the higher steric hindrance of foulants within or on the structure of the membrane which was a result of the adsorption of organic matter onto the surface of the membrane. Additionally, it was postulated to have been due to the enhanced interactions and electrostatic repulsion between solutes and foulants.

It is important to note that these studies have limitations, as they primarily used deionized water spiked with common PFASs such as PFOA and PFOS. This approach may not accurately reflect the behaviour of other PFAS compounds. Additionally, the use of deionized water does not accurately represent the characteristics of real water matrices. To address these limitations, future studies should incorporate a wider range of PFAS compounds to ensure the applicability of the results to various PFASs. Using real water matrices instead of deionized water will provide more accurate insights into the behaviour of PFASs in practical settings. Further, utilizing natural organic matter from actual water sources will better represent the conditions and challenges encountered in real-world water treatment scenarios. This approach will enhance the relevance and reliability of the findings.

**Figure 9:**
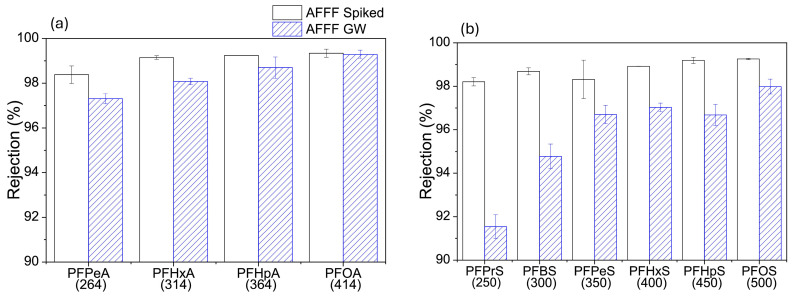
Rejection of (**a**) PFCAs and (**b**) PFSAs using NF membrane in spiked AFFF and groundwater solutions (Reproduced with permission from Ref. [8]).

Although organic matter has been found to increase the retention of PFASs, in some cases, the adsorption of organic matter has had the opposite effect. Liu et al. [8], obtained contrary results where a decrease in the rejection of short-chain PFASs in aqueous film-forming foam (AFFF) groundwater was observed, which was attributed to organic matter being present in groundwater (Figure 9). Notably, this was previously shown to lower the surface charge of the membrane, leading to reduced repulsive forces between PFAS molecules and the membrane surface. Consequently, the repulsive forces are what are largely responsible for the rejection of short-chain PFASs. The decrease in PFAS rejection can also be attributed to an increase in concentration polarization, which occurs when PFAS molecules accumulate on the surface of the membrane, forming a cake layer. This layer then creates a concentration gradient that reduces filtration efficiency. For instance, a study confirmed that the rejection of PFAS molecules decreased from 99.3% to 95.3% after fouling with SA, due to foulant-enhanced concentration polarization [31]. Therefore, while both cases are valid regarding the rejection of PFASs in the presence of organic matter, selecting materials that do not promote membrane fouling is crucial for extending membrane lifespan and reducing costs.

#### 3.1.2. Solute Concentration

The concentration of PFAS solutes in feedwater can significantly impact the fouling and permeate flux of the membrane as well as affect the rejection (*R*) of PFASs in water, which is calculated by comparing the concentration of PFASs in the feed (*C_f_*) and permeate (*C_p_*) solutions according to Equation (6)

(6) $$R=1-\frac{C_{f}}{C_{p}}\times \;100\%$$

**Figure 10:**
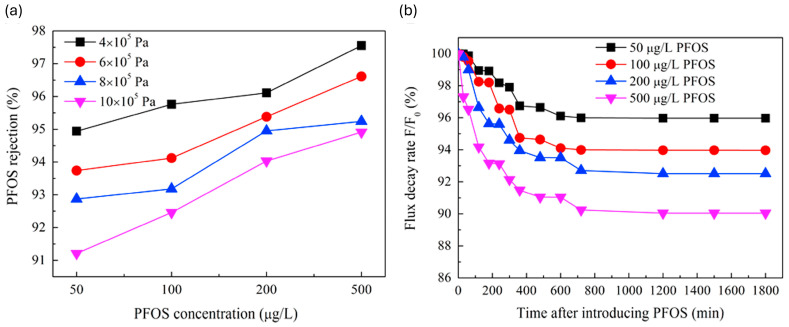
(**a**) Rejection of PFOS with varying concentrations. (**b**) Influence of PFOS concentration on the Flux decay rate (F/F_0_): F_0_ is the pure water flux; F is the flux at a specific moment in time. (Reproduced with permission from Ref. [62]).

Hang et al. [61] tested the rejection of PFOA using two commercial membranes (NF270 and NF90) where NF90 had an almost 100% rejection of PFOA at various concentrations (200–800 mg/L), which was much higher than that of NF270, due to the difference in the molecular weight cutoff (MWCO). The rejection of PFOA was enhanced by increasing its concentration in the feed due to an increase in the adsorption of PFOA on the membrane surface. The adsorbed PFOA altered the surface charge of the membrane, leading to a greater repulsion of PFOA and, subsequently, a higher rejection of PFOA [61]. These results corroborate the findings of Wang et al. [62], wherein the rejection of PFOS increased as the concentration of PFOS increased, as illustrated in Figure 10a. The concentration of PFOS caused an accumulation on the membrane surface and within the pores, leading to a change in the membrane’s surface charge. This alteration caused electrostatic repulsion between PFOS and the membrane surface, which in turn enhanced the rejection of PFOS. 

Although high concentrations of PFASs lead to high rejections, the water flux is negatively impacted, leading to reduced productivity and increased energy requirements, as shown in Figure 10b.

#### 3.1.3. Transmembrane Pressure

Transmembrane pressure (TMP) plays a significant role in the rejection of PFASs. Along with rejection efficiency, TMP also affects the fouling and integrity of the membrane and permeate flux, which is described by Equation (7).

(7) $$J_{w}=\frac{V}{A\times t\times P}\;$$

whereby *V* is the volume (L) of water that is collected, *P* is the operating pressure (bar), *A* is the effective filtration area (m^2^) of the membrane and *t* is the time needed to collect water (h).

**Figure 11:**
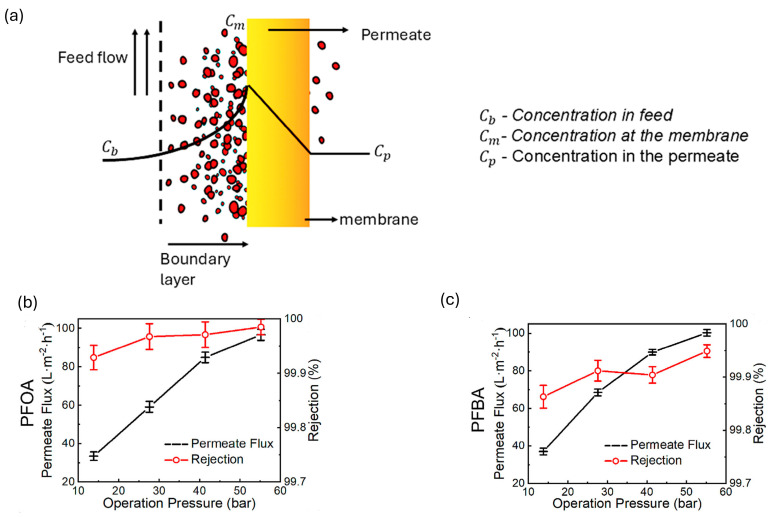
(**a**) Schematic representation of the accumulation of molecules on surface of membrane (this work). (**b**,**c**) Rejection and permeate fluxes of PFOA and PFBA at various operation pressures [57].

As TMP increases, the driving force for water permeation through the membrane also increases. The rejection of PFASs, particularly long-chain PFASs, also increases due to the tightening of the membrane structure which reduces the pore size of the membranes. However, it has been observed that during high-pressure filtration, PFAS molecules can form micelles (clusters of PFAS molecules) that aggregate on the membrane surface. Micelle formation can block membrane pores and reduce permeate flux. Additionally, it can result in severe fouling and contribute to CP, where solutes accumulate on the membrane surface, further decreasing filtration efficiency and negatively affecting overall system performance (Figure 11a) [56,61]. For instance, Ma et al. [57] observed the effect of TMP on the permeate flux and rejection of PFBA and PFOA. They found that when the TMP exceeded 40 bar using a commercial BW30-2540 RO membrane, permeate flux increased but not linearly. This was due to the compression of the membrane which reduced the effective pore size and water transfer channels, as depicted in Figure 11b,c. This compression enhanced the membrane’s rejection ability towards PFASs. Further, higher TMP increased water permeate flux, leading to a dilution effect that lowered the concentration of PFASs in the permeate. This effect combined with the solution–diffusion mechanism where solute flux remains independent of pressure results in higher PFAS rejection. The well-defined pore size and MWCO of the RO membrane ensure stable rejection performance even under increased pressures.

In a similar vein, another study found the rejection rate of PFOS to decrease as the pressure increased from 4 bar to 10 bar. This was ascribed to the concentration polarization which is high at elevated pressures and reduces the rejection of PFOS as a result. The primary mechanism responsible for the rejection of PFOS was found to be electrostatic repulsion between the PFOS molecules and the membrane since the MWCO of the PMIA membrane was higher than that of PFOS (538 Da); therefore, concentration polarization hindered this mechanism, thus reducing rejection [62].

#### 3.1.4. Solution pH

**Figure 12:**
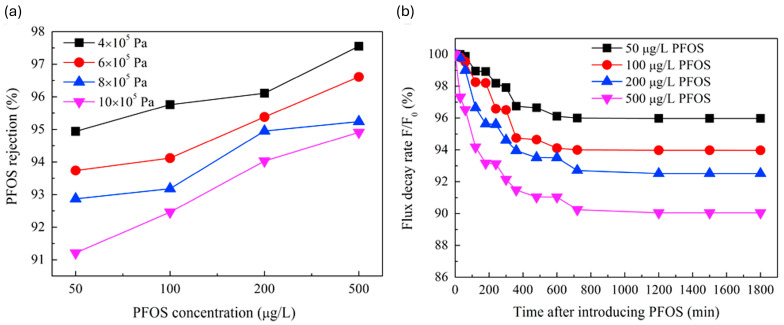
(**a**) Influence of pH on rejection of PFOS. (**b**) Zeta potentials of PMIA membrane at various solution pHs (reproduced with permission from Ref. [62]).

The pH level changes the membrane surface charge and the speciation of PFAS molecules into their conjugate base and acid forms which influences the interaction between the membrane surface and PFAS molecules. Under neutral and alkaline pH conditions, the majority of PFAS molecules are anionic [31], thus leading to electrostatic repulsion from RO or NF membrane surfaces which are also generally negatively charged [63,64,65]. The effect of pH on the separation efficiency of PMIA NF hollow fibre membranes in removing PFOS was investigated by Wang et al. [62]. From the findings at lower pH levels, the PMIA membrane exhibited a positive charge and a negative charge at high pH levels, whilst PFOS was negatively charged throughout the studied pH range (pKa ≈ −4). Therefore, the removal of PFOS was expected to vary with changes in solution pH. At a pressure of 4 bar, the rejection of PFOS increased from 91.17% to 97.49% as the solution pH changed from 3.2 to 9.5, as observed in Figure 12a.

Looking at the zeta potentials of the membrane in Figure 12b, it was observed that the membrane surface charge became more negative as the pH increased. This was due to the presence of dissociable carboxylic groups on its surface. This then resulted in stronger electrostatic repulsions between the membrane surface and PFOS molecules with increasing pH [61]. 

The effects of pH on the rejection of perfluorohexanoic acid (PFHxA) by five commercial polyamide NF (NF90 and NF270) and RO (BW30, XLE and SW30XLE) membranes have been demonstrated by Soriano et al. [66]. At neutral pH (7.1), all membranes had a high rejection (>99%) except NF270 (96.2%), while at acidic pH (3.5), the rejection drastically decreased to 86.9% for the NF270 membrane, while the other four membranes still had >95% rejection. This observation was attributed to the following: Firstly, PFHxA is anionic at the studied pH 3.5 and 7.1 (pKa ≈ −0.136) [30], and the membranes were negatively charged as well; therefore, electrostatic repulsion was experienced, which led to high rejections. Secondly, the isoelectronic point of the NF270 membrane was the closest to the studied acidic pH of 3.5 which eliminated the electrostatic repulsion experienced by PFHxA to the membrane surface. Thirdly, the MWCO of NF270 was the closest to the size of PFHxA, which limited the steric hindrance experienced by PFHxA on the membrane. The other four membranes had a much lower MWCO compared to the size of PFHxA, hence being less affected by the change in pH as the rejection was due to both steric hindrance and electrostatic repulsion.

#### 3.1.5. Ionic Strength

The ionic strength of the feed solution affects the rejection of PFASs as it influences the interactions between the membrane surface, the ions in the solution and PFASs [60,61]. At high ionic strength, the concentrations of ions such as Ca^2+^ and Na^+^ are also high. For negatively charged membranes, these ions can neutralize the surface of the membrane, leading to reduced repulsive forces being experienced by PFASs and the membrane surface. 

In a study conducted by Wang et al. [60], the rejection of PFBS (a short-chain PFAS) using an NF poly (piperazineamide) membrane decreased from 48.9% to 20.5% as the ionic strength went from 0 to 100 mM in the feed solution. This decrease was attributed to the reduction in the electrostatic repulsion observed between the membrane surface and PFBS. This was due to increasing the concentration of competing ions (Na^+^) on the surface of the membrane. Considering that the size of the PFBS molecules (0.715 nm) is smaller than the average membrane NF pore size (0.912 nm), the PFBS molecules were able to pass through the pores of the membrane with ease.

**Figure 13:**
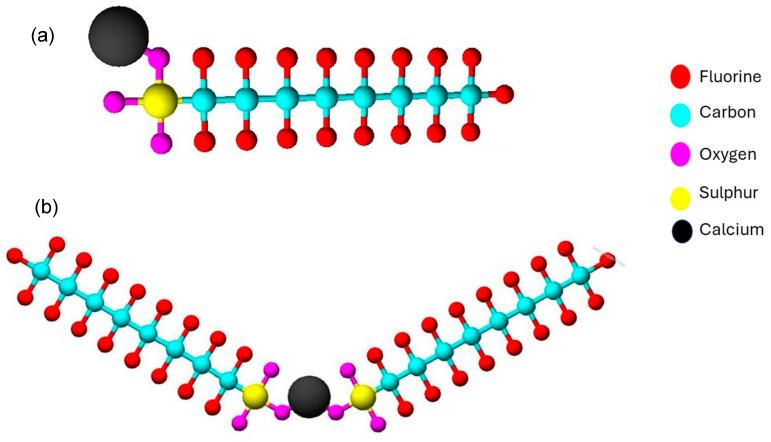
(**a**) Formation of CF_3_(CF_2_)_7_SO_3_Ca due to electrostatic interaction between Ca^2+^ and negatively charged sulfonate group; (**b**) formation of CF_3_(CF_2_)_7_SO_3_ −Ca− O_3_S(CF_2_)_7_CF_3_ through linkage of two PFOS molecules to Ca^2+^.

Additionally, the ions present can form bridging interactions with PFASs in solution, leading to an increase in the size of PFAS molecules which then enhances the rejection of PFASs. For instance, Zhao et al. [67] demonstrated the rejection of PFOS to increase from 94% to >98% when the concentration of Ca^2+^ was increased from 0.1 mmol/L to 2.0 mmol/L. This enhanced rejection was attributed to the formation of CF_3_(CF_2_)_7_SO_3_Ca^+^ and CF_3_(CF_2_)7SO_3_ − Ca − O_3_S(CF_2_)_7_CF_3_, as shown in Figure 13a,b, which increased the size of the PFOS molecules, thus enhancing size exclusion. Further, Ca^2+^ neutralized both the negatively charged NF270 membrane and the PFOS anions which enhanced the adsorption of PFOS on the membrane surface, causing a dense layer to form on the surface, resulting in a reduced flux [67,68].

#### 3.1.6. Membrane Characteristics

##### Membrane Pore Size

The pore size and MWCO of membranes are crucial factors influencing their ability to reject PFAS chemicals effectively. Generally, the MWCO of membranes should be smaller than that of PFAS molecules to ensure effective rejection. Nanofiltration (200–1000 Da) and RO (150–300 Da) membranes have been noted for their effective rejection of PFASs in water due to their smaller pore size and MWCO; however, this is mainly observed for long-chain PFASs such as PFOA (414.07 g/mol) and PFOS (500.126 g/mol) [69,70]. 

**Figure 14:**
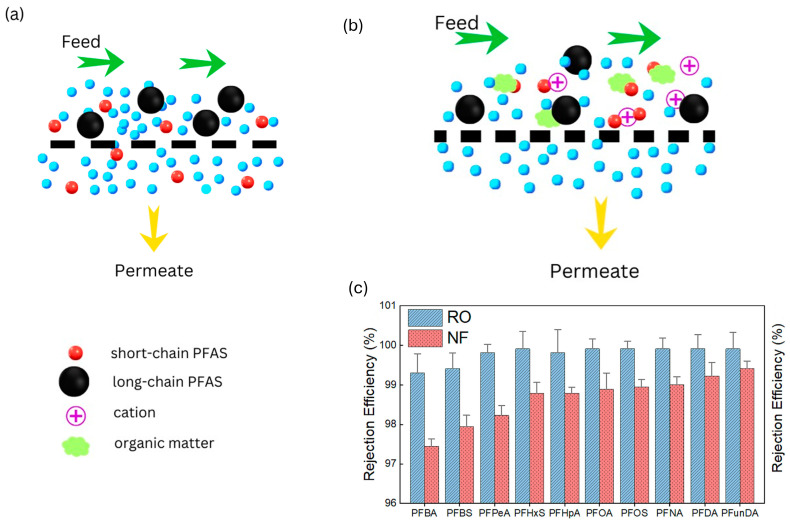
Schematic representation of the rejection proficiency of NF membrane for long- and short-chain PFASs (**a**), effect of organic matter and cations on rejection of long- and short-chain PFAS molecules (**b**) (this work) and (**c**) rejection proficiency of RO and NF membranes for long- and short-chain PFASs [57].

Short-chain PFASs typically have a lower MWCO (i.e., 114.02 g/mol for trifluoroacetic acid (TFA)); therefore, they can pass through membranes that have a higher MWCO compared to short-chain PFASs, as observed in Figure 14a. Consequently, other mechanisms besides size exclusion (such as electrostatic interactions and hydrophobic effects) play a more significant role in the rejection of PFASs. For instance, Ma et al. [57] evaluated the rejection of 10 different PFASs with different chain lengths using NF and RO membranes. Long-chain PFASs such as Perfluorodecane sulfonic acid (PFDA) and Perfluoroundecanoic acid (PFUnDa) had higher rejections than short-chain PFASs including PFBA and PFBS (Figure 14c).

The existence of Ions such as Ca^2+^ and Mg^2+^ can bind to PFASs, resulting in the formation of bridging interactions which are larger in size, thus enhancing the rejection of PFASs, as observed in Figure 14b [50]. The same was observed when organic matter such as HA, BSA and SA were present [57,58]; a cake layer formed which further decreased the membrane pore size while also enhancing the rejection of PFASs.

##### Membrane Surface Charge and Hydrophobicity

The membrane surface charge affects the electrostatic interaction between the membranes and PFAS molecules, which influences their rejection. Zeta potential is commonly used to measure the charge of a membrane surface which reflects the strength of the electrostatic interactions between PFAS molecules and the membrane surface. Generally, a higher negative potential corresponds to a more negatively charged surface which enhances electrostatic repulsion. 

**Figure 15:**
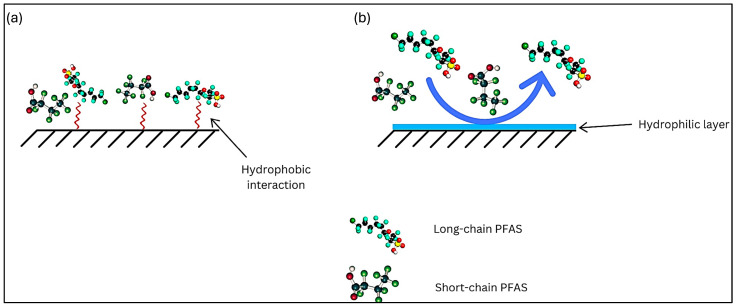
Schematic representation on the interaction of PFASs with membrane with (**a**) hydrophobic and (**b**) hydrophilic surface.

Recently, Zeng et al. [70] examined the rejection of PFHxA using various commercial membranes, namely RO (NTR-759 HR), NF (NTR-7410 and NTR-7450) and UF (UP020 and UH030). All membranes exhibited negative charges to varying degrees, signifying that electrostatic repulsion was one of the mechanisms responsible for the rejection of PFHxA. From Figure 15, it is observed that the more negative the zeta potential, the higher the rejection of PFHxA tends to be. PFHxA dissociates into PFHxA ions (CF_3_(CF_2_)_4_COO^−^) in water, leading to charge repulsion occurring between negatively charged membranes and PFHxA ions taking place. 

Another important factor to consider is the hydrophobic or hydrophilic nature of membrane surfaces, which not only influences the rejection of PFASs but also affects membrane fouling. PFASs are inherently hydrophobic due to the C-F bonds in their structure, with hydrophobicity increasing as the chain length grows. Therefore, using membranes with a hydrophobic surface enhances the hydrophobic interactions between the membrane surface and PFASs such as PFOA and PFOS which results in the adsorption of PFASs onto the membrane surface, leading to fouling (Figure 15a). 

On the contrary, membranes with a hydrophilic surface enhance PFAS rejection while reducing membrane fouling, as PFAS molecules and other compounds are less likely to adsorb onto the surface (Figure 15b) [71]. To reduce PFAS adsorption onto the surface of the membrane as a result of hydrophobic interactions, modulating surface hydrophilicity is key. This can be conducted by incorporating different functional materials (i.e., nanomaterials, additives or hydrophilic monomers) into the polymer matrix during the processes of chemical functionalization. It is also critical to study the physicochemical properties and long-term stability of the modified membrane surface [70]. 

Wu [72] studied the rejection of PFOA and PFOS using a polyamide NF membrane with a MXene-TiO_2_ (MXT-NFM) interlayer. The interlayer possessed abundant functional groups which were hydrophilic such as epoxy and hydroxyl groups. This increased the hydrophilicity of the membrane which helped alleviate membrane pore clogging and enhance permeability. The rejection of PFOS and PFOA was slightly enhanced in comparison to the pristine membrane due to an elevated negative charge on the membrane surface which enhanced the repulsive forces. The water contact angle for MXT-NFM and the pristine membrane was 22.4° and 44.9°, respectively, indicating the membrane’s improved hydrophilicity with the incorporation of MXT-NFM. The membrane showed good anti-fouling performance in the presence of Ca^2+^, HA and PFAS molecules with the rejection of PFOA and PFOS using the MXT-NFM membrane being 95.48% and 99.73% at pH 10, respectively. This high rejection efficiency is attributed to the repulsion between the negatively charged PFOA and PFOS molecules and the negatively charged membrane at this pH [72]. 

Conversely, Chen et al. [73] looked at direct contact membrane distillation (DCMD) which is a method that employs a hydrophobic membrane that is porous for liquid separation to remove and concentrate perfluoropentanoic acid (PFPeA) from synthetic water using polytetrafluoroethylene (PTFE) membranes. The membrane demonstrated a progressive increase in flux (from 17 to 43 kg m^−2^ h^−1^) and a decrease in PFPeA rejection (from 85% to 58%) as the temperature of the feed increased from 50 °C to 70° C. However, the adsorption of PFPeA molecules onto the PFTE membrane surface due to the hydrophobic–hydrophobic interactions caused the membrane to experience severe fouling. The fouling increased with increasing temperature corresponding to the elevated rate of evaporation in the feed. To combat fouling, our recommendation is to further investigate materials which enhance the hydrophilicity of the membrane to combat the adsorption/hydrophobic interactions resulting in the adsorption of PFASs onto the surface of molecules and will enhance the electrostatic repulsion between the surface and PFAS molecules. 

##### Membrane Material

The type of membrane material greatly influences its performance in rejecting PFASs. Multiple membrane materials have distinct properties which influence their interactions with PFAS molecules which results in their different rejection efficiencies. For instance, polymeric membranes including polyamide (PA), polyvinylidene fluoride (PVDF) and polyethersulfone (PES) are commonly used for the rejection of PFASs due to their ability to form tight molecular structures and chemical resistance [71]. Polyamide membranes, commonly used in NF and RO processes, have a dense structure and small pore sizes that lead to high rejection rates for PFASs by hindering the passage of PFAS molecules [71]. These membranes can operate effectively across a wide pH range, making them suitable for treating multiple types of feed water, including drinking water, wastewater and landfill leachate. Despite their efficiency in PFAS removal, these membranes are subject to fouling and mineral scaling due to PFAS molecules adsorbing onto the surface of the membrane which lowers permeate flux [74]. 

**Figure 16:**
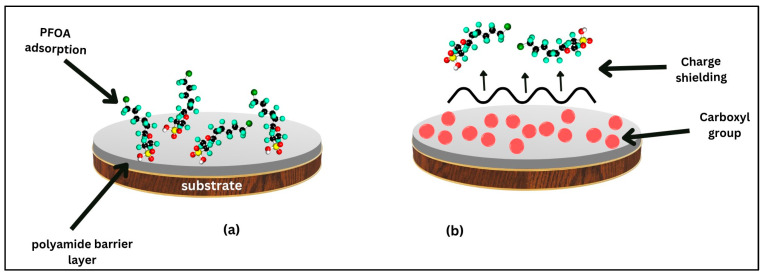
Schematic diagram showing the adsorption of PFOA onto polyamide barrier layer (**a**); electrostatic repulsion between polyamide barrier layer modified with carbonyl groups and PFOA (**b**).

To address this issue, efforts to make the membrane surface more negative have been explored. This lessens the scaling and fouling experienced by the membrane because of the elevated electrostatic repulsion between the membrane surface and PFAS molecules, as illustrated in Figure 16. Boo et al. [75] developed an NF membrane with a selective polyamide surface layer characterized by a negative surface charge and a loose nanoporous structure. This design aimed to aid the transfer of scale-forming cations while enhancing the rejection of PFOA. The membrane integrated onto a PES support was synthesized via interfacial polymerization using trimesoyl chloride and a combination of bipiperidine and piperazine. The NF membrane showed significant PFOA retention (~90%) while enabling the high level of permeation of scale-forming cations like calcium. The presence of carbonyl groups on the polyamide surface layer played a crucial role as a charge shield, preventing PFAS molecules from adsorbing to the membrane surface [75].

Therefore, when selecting materials to fabricate membranes, it is imperative to consider the composition of the feedwater that will be treated by the membranes. As discussed, the presence of ions including Ca^2+^ and organic matter could decrease the electrostatic repulsion between the membrane surface and PFASs by binding to the membrane surface and PFASs, leading to lower rejection efficiency.

### 3.2. Membranes Used for Rejection of PFASs

#### 3.2.1. Ceramic Membranes

Ceramic materials have been widely used, owing to their excellent thermal stability, mechanical strength and chemical stability which allow them to be coupled with AOPs such as photocatalysis to effectively mineralize PFASs [75]. Ceramic membranes have shown great advantages in the treatment of water, separation of high-value-added products, treating viscous and particulate fluids and working in a harsh environment unlike polymeric membranes. Ceramic ultrafiltration (UF) and microfiltration (MF) membranes have been commercialized and used in the treatment of various wastewaters including oily water, paper mill effluents and pharmaceutical wastewater and the treatment of organic matter in drinking water treatment plants [76,77,78,79]. The use of ceramic membranes in the removal of PFASs has also been investigated [80]. 

Despite their excellent stability, ceramic membranes face several challenges that make them unsuitable for manufacturing RO and NF membranes. These challenges include difficulties in controlling pore sizes, inherent brittleness, surface roughness leading to fouling issues and the complexity and high cost of manufacturing processes. Consequently, ceramic membranes have not been as effective in removing PFASs due to their larger pore sizes. However, ceramic membranes can be coupled with other processes to efficiently remove PFASs, leveraging their strengths while compensating for their limitations. 

For example, Liu et al. [81] fabricated a functionalized ceramic membrane using BFO for the removal and degradation of PFOA. The ceramic membrane demonstrated good stability and resistance to irradiation and radical attack. Initial experiments with the pristine membrane showed it to only remove about 2% of PFOA in solution, indicating that the size exclusion and repulsion of PFOA were negligible. However, a decrease in the concentration of PFOA was observed in the permeate when the coated BFO membrane was used. The initial decrease in PFOA concentration was attributed to its adsorption onto the membrane. However, as the reaction proceeded and adsorption reached equilibrium, the concentration of PFOA in the permeate began to rise.

When the membrane was subjected to irradiation, the concentration of PFOA further decreased in the permeate, signalling the degradation of PFOA which quickly increased when irradiation was turned off. The coated membranes were found to remove 65.9% of PFOA using microwave irradiation.

In 2019, Murray et al. [82] utilized a ceramic membrane and superfine powder activated carbon (SPAC) to remove a range of PFASs in AFFF-contaminated water. SPAC acted as an adsorbent and reduced fouling due to the adsorbent acting as a thin protection layer which prevented foulants such as PFASs and organic matter from encountering the membrane. An analysis of the permeate found less PFASs including long-chain PFASs such as perfluorooctane sulfonamide (FOSA) being detected, but short-chain PFASs such as perfluoropropane sulfonamide (FPrSA) were present which shows the inefficiency of the system in removing short-chain PFASs. Additionally, Zhou et al. [83] synthesized a silica membrane coated with Fe_3_O_4_ nanoparticles with fluorinated groups (Fe_3_O_4_@SiO_2_-NH_2_&F_13_) for the rejection of PFOA, PFHpA, PFNA, PFTA, PFHxS PFUnDA, PFDoDA and PFDA. The reported experimental conditions were as follows; pH 3, volume 1 L and concentration 0.5–50 ng/L. The results showed that Fe_3_O_4_@SiO_2_-NH_2_&F_13_ had a strong affinity to PFAS molecules due to fluorine–fluorine (F-F) interactions between the membrane and PFASs. These interactions allowed the membrane to remove 86.29% of PFASs which was higher compared to powdered activated carbon (PAC) which they found to remove 58.61% in surface waters. To simulate real water matrices, samples were spiked with HA. Under these conditions, the membrane removed 75% of PFASs. Meanwhile, PAC’s removal efficiency dropped significantly to 12.32%, highlighting the membrane’s superior selectivity.

#### 3.2.2. Polymeric Membranes

Polymeric membranes used in NF and RO are primarily fabricated from materials like polyethersulfone (PES), polyvinylidene fluoride (PVDF) and polysulfone. These materials are ideal due to their stability and the ability to precisely control pore sizes (which is crucial for effective PFAS rejection) [84]. Most existing water treatment membranes are made of organic polymers, which offer advantages over inorganic membranes due to their easier processing and suitable robustness. Commercially available RO and NF membranes, predominantly used for PFAS removal in water purification, are typically thin-film composite (TFC) polyamide (PA) membranes. Flexibility in polymer chemistry allows for surface modifications to enhance fouling resistance and selectivity, further improving the efficacy of these membranes in treating contaminated water sources [84].

Hang et al. [61] studied the rejection of PFOA using commercial nanofiltration NF90 and NF270 membranes. Both membranes had a polyamide selective layer but different MWCOs; NF90 had an MWCO of 90–200 Da, while NF270 had an MWCO of 155–300 Da. When observing the rejection of PFOA at various concentrations, NF90 showed higher rejections than NF270. The performance of the two membranes was attributed to the NF90 MWCO compared to that of the NF270 membrane, so size exclusion was enhanced. Zeng et al. [70] also highlighted the efficiency of the commercial membranes in rejecting PFASs using the NF270 and NTR-7450 membranes. The NF270 membrane featured a polyamide selective layer with an MWCO of 200 Da, while the NTR-7450 membrane had a sulfonated polyethersulfone selective layer with an MWCO of 600–800 Da. Both membranes efficiently rejected PFHxA, with NF270 rejecting 97% of the molecule and NTR-7450 rejecting 96%. The main mechanism responsible for the rejection was the repulsion between the PFHxA molecules and the membrane surface. NTR-7450 was not affected by varying the pH of the solution as the sulfonated polyethersulfone selective layer also featured negatively charged sulfonated groups (−SO_3_H) which were negative throughout all pH values. 

These findings emphasize the importance of membrane characteristics in optimizing PFAS removal, with size exclusion and electrostatic interactions being crucial for achieving high rejection efficiencies. Despite these advancements, membrane fouling continues to be a significant issue. Effectively removing multiple pollutants such as PFOA and NOM from water using NF remains a challenge. To address these challenges, researchers are exploring the incorporation of various nanomaterials to enhance NF performance and mitigate issues related to fouling and overall efficiency, as shown in Table 3. Fang et al. [85] fabricated an adsorptive hydrophilic UF membrane using polyacrylonitrile (PAN) polymer for the removal of PFOS. The membrane was modified with amine groups to further enhance hydrophilicity which resulted in the minimization of fouling experienced by the membrane. The membrane efficiently removed 98% of PFOS with minimal fouling experienced over various cycles, highlighting its durability and stability.

In another study, Jin et al. [86] conducted a study focusing on the fabrication of low-cost membranes using amyloid fibrils derived from β-lactoglobulin for the removal of PFASs. These membranes were produced using whey protein, offering a sustainable and environmentally friendly alternative to traditional membrane fabrication methods. The performance of these membranes was evaluated using river water samples collected near a large fluoropolymer production site in China. The results showed a sizable reduction in PFAS concentrations: PFOA levels decreased from 327 μg/L to 241 μg/L, and PFBA levels dropped from 102 μg/L to 3.65 μg/L after filtration. From an environmental and economical perspective, amyloid–carbon hybrid membranes were found to outperform traditional nanofiltration membranes in several ways. The fabrication process of these protein-based membranes is solvent-free and energy-efficient, unlike conventional polymeric membrane fabrication which involves the release of toxic organic solvents and high energy consumption. Moreover, the operational energy demands of nanofiltration add further environmental stress, whereas the protein-dominant processes used in creating amyloid fibril membranes mitigate these issues, making them a greener and more sustainable option for PFAS removal.

#### 3.2.3. Two-Dimensional Material Membranes

Two-dimensional nanomaterials, such as MXene, graphene oxide (GO) and boron nitride (BN), have recently gained attention for their use in membrane modification due to their large surface area, hydrophilicity and excellent mechanical properties [87,88,89,90,91]. MXene (Ti_3_T_2_C_x_) membranes have demonstrated immense potential for PFOA removal in separation processes, owing to their negatively charged surfaces, high hydrophilicity, robust mechanical strength and ultrahigh flux. Their tuneable interlayer channels also provide a significant advantage over conventional nanofiltration membranes by enhancing the balance between permeability and selectivity for PFOA removal. This makes MXene membranes a promising candidate for improving the efficiency of water purification systems targeting persistent contaminants like PFOA [87,88,89].

**Table 3:** Modified NF membranes for the removal of PFASs.

Membrane	Selective Layer	PFAS	Operation Parameters	Removal Efficiency	Remarks	Reference
Graphene oxide	Polyethyleneimine (PEI)	PFOA	[PFOA] = 50 ppm pH = 7.0 Temp = 25 °C TMP = 1 bar Crossflow rate = 0.25 L/min	96.5%	PEI improved mechanical stability and enhanced retention	[92]
Polysulfone (PSF) hollow fibre support	Polyamide–MXene nanosheets	PFOS	[PFOS] = 2 ppm pH = 5.5 Temp = 25 °C TMP = 4.5 flow rate = 0.3 mL/min	96%	MXene nanosheets modified surface charge and morphology of membrane surface, thus improving rejection of PFOS	[93]
NF hollow fibre membrane	Poly(*m*-phenylene isophthalamide)	PFOS	[PFOS] = 100 μg/L pH = 5.5 Temp = 25 °C TMP = 4 bar flow rate = 1.50 L/min	99.40%	Donan exclusion and steric hindrance were responsible for efficient rejection of PFOS	[62]
Thin-film composite NF with Hyaluronic (HA) interlayer	Polyamide (PA)	PFHxS	[PFHxS] = 100 μg/L pH = 5.5 Temp = 25 °C TMP = 4 bar	93.4%	HA layer resulted in thinner PA layer and improved hydrophilicity, resulting in higher permeability	[94]
Aluminium oxide hydroxide	Linear fluorinated silanes	PFOA PFOS	[PFOA] = 390 ppt [PFOS] = 860 ppt pH = 7.5 Temp = 25 °C	>90%	Linear fluorinated silanes decreased pressure drop in γ-AlOOH filter whilst maintaining 99.9% rejection of PFOA and PFOS	[95]

To further optimize the balance between permeability and rejection, Xu et al. [87] explored the performance of MXene/CNT membranes under various conditions for PFOA removal. Their study demonstrated rejection rates as high as 91% and high permeance when assessed with real water samples collected from the Changdang Lake waterworks in China, highlighting the practical effectiveness of MXene-based membranes in real-world water treatment applications. Additionally, Ma et al. [88] further showcased the selectivity of MXene membranes in the rejection of PFHxS (96.85%) and PFHxA (93.35%) while maintaining high permeability which was attributed to MXene’s hydrophilic functional groups that enhanced the hydrophilicity of the membrane surface. Additionally, the presence of electron-rich atoms facilitated the transport of water molecules. The increased electronegativity of the negatively charged MXene surface also proved beneficial for the separation of negatively charged PFAS compounds. 

GO membranes, on the other hand, have demonstrated high selectivity for PFOA removal in wastewater streams. However, a study by Khorramedal et al. [90] revealed challenges such as membrane swelling during preparation and reduced selectivity due to large interlayer galleries. To address this, the membranes were modified with poly (diallyl dimethyl ammonium chloride) (PDDA) and poly (styrene sulfonate) (PSS), which reduced membrane swelling and adjusted the interlayer galleries. These modifications significantly enhanced the hydrophilicity of the membranes, as indicated by a reduction in the contact angle from 63.4° for unmodified GO to 35.2° for PSS-GO and 38.9° for PDDA-GO membranes. The modified membranes achieved water fluxes of 83.1 L/m^2^h for PSS-GO and 80.5 L/m^2^h for PDDA-GO, representing a 325% increase compared to the pristine GO membrane (18.63 L/m^2^h). The introduction of high surface charges and electron-rich nanosheets not only reduced the interlayer spacing in the modified membranes but also improved surface hydrophilicity, leading to enhanced PFOA retention and greater water permeability.

## 4. Photocatalytic Membrane Reactors

The integration of photocatalysis and membrane separation for the efficient removal and degradation of PFASs has garnered attention due to the ability of the systems to overcome the drawbacks associated with either technology. The difficulties associated with separating, removing and reusing photocatalysts after treatment may limit the large-scale application of photocatalytic degradation. These challenges have led to the exploration and adoption of technologies like photocatalytic membrane reactors [90]. In these reactors, photocatalysis is utilized for the degradation of pollutants, whilst the membrane separates the resultant products of photocatalytic degradation as well as the photocatalyst depending on the configuration of the system [96]. 

The most used membranes used in these reactors include MF, UF and NF, and based on the configuration of the system, two main types can be noted, namely (1) one with a photocatalyst immobilized in/on the membrane and (2) another with a photocatalyst in suspension [97,98,99,100]. Photocatalytic membrane reactors are efficient in degrading various pollutants including pharmaceuticals and dyes; therefore, the advancement of photocatalytic membrane systems has become a widespread and effective method for the treatment of contaminated water [96,101]. However, more research still needs to be conducted on the remediation of PFASs. 

### 4.1. Photocatalytic Membrane Reactors with Suspended Photocatalysts (Slurry Reactors)

The concept of slurry reactors involves suspending photocatalyst particles (such as TiO_2_) in a solution and circulating them through a membrane module. This configuration allows for direct interaction between PFAS molecules and the photocatalyst in the solution, thus enhancing degradation efficiency while simultaneously facilitating membrane filtration for treated water recovery [100,102]. A notable benefit of this system is that the PFAS molecules can effectively interact with the photocatalyst because of its larger surface area which is limited in photocatalytic membranes.

Research indicates that the kinetics of this process align with the Langmuir–Hinshelwood (L-H) model, as described by Equation (8), which outlines the interaction of reactants with the surface of the photocatalyst.

(8) $$ro=-\frac{dC}{dt}=\frac{kKC}{1+KC}$$

whereby ro is the degradation rate of the reactants, k is the reaction rate constant, K is the adsorption equilibrium constant and C is the concentration of PFASs [103].

In the case where K << 1, Equation (8) can then be simplified further to a pseudo-first-order kinetic model

(9) $$-\frac{dC}{dt}=kKC=k_{obs}C$$

Equation (9) can also be further simplified to Equation (10)

(10) $$-\ln\frac{C}{C_{o}}=k_{obs}t$$

**Figure 17:**
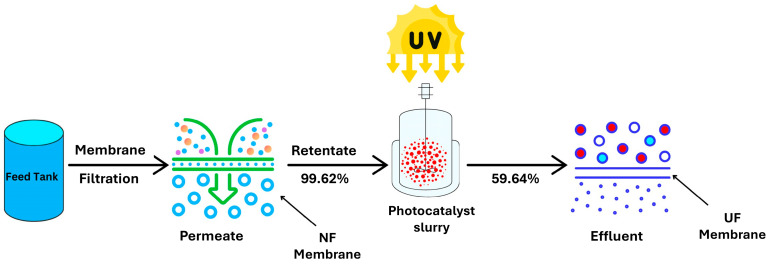
Schematic diagram showing the removal of PFOA using NF membrane–UV hybrid system.

Given that PFASs are typically present at trace concentrations in contaminated water, in ppt or ppb, subjecting water directly to photocatalysis would not be practical. Hence, starting with membrane separation to concentrate PFASs and then applying photocatalysis to the concentrated stream is considered a more efficient and viable approach. Boonya-atichart et al. [100] studied the performance of a slurry system for the separation and degradation of PFOA from spiked deionized (DI) water and groundwater. The PFOA feed solution initially went through a NF membrane, with an MWCO of 200 Da, which rejected 99% of PFOA due to size exclusion, leading to a concentrated stream from both the synthetic water and groundwater. The concentrated stream was then fed to a UV tank that had zero valent iron nanoparticles (nZVI) as the photocatalyst and was then irradiated with UV light which initiated the degradation of PFOA, as shown in Figure 17. The reactor chamber was equipped with a circulation pump that ensured the continuous flow of the PFAS-contaminated feed water, allowing for consistent interaction between the membrane, PFAS molecules and the photocatalyst.

Low degradation rates were observed for both the groundwater (59.64%) and spiked DI water (73.95%) due to a lack of a mixer in the UV tank to sufficiently distribute the nanoparticles to allow for adequate interaction between the nanoparticles and PFOA molecules. Additionally, the positioning of UV light limited the exposure of the solution to UV irradiation, thus hindering the degradation process. To improve the system’s performance, future studies should focus on incorporating a mixer to ensure uniform nanoparticle distribution and optimizing the placement of UV light sources for a better coverage and fouling of membranes and long-term operating costs. Furthermore, testing various UV intensities and nanoparticle concentrations could enhance photocatalytic efficiency and provide insights for real-world applications.

**Figure 18:**
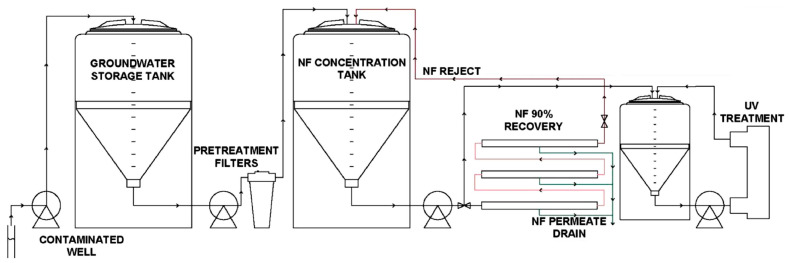
Flow diagram of NF/UV–sulphite pilot system for treatment of groundwater (reproduced with permission from Ref. [104]).

In 2021, Liu et al. [104] conducted a pilot-scale treatment of PFASs from AFFF-contaminated groundwater using a hybrid NF and UV–sulphite system. Initially, water passed through the polymeric NF membrane, leading to the rejection of PFASs and a concentrated stream being produced for further treatment using a UV–sulphite system to degrade PFASs, as illustrated in Figure 18. The study evaluated 12 different types of PFASs, ranging from short-chain perfluoropropane sulfonate (PFPrS) to long-chain PFOS, with PFAS rejection rates ranging from 88% to over 95%. The high rejection rate was attributed to a combination of size exclusion and electrostatic repulsion. At pH 7.3 (pH of the groundwater), the negatively charged NF membrane resulted in the repulsion of anionic PFAS molecules, while size exclusion was effective because the MW of the PFAS molecules used (250–500 g/mol) exceeded the MWCO of the NF (180 g/mol) membrane. 

Following membrane separation, the concentrated stream from the NF membrane was subjected to UV irradiation at 254 nm in the presence of 10 mM sulphite for 30 consecutive days to monitor the stability of the system. The sulphite concentration enhanced the production of hydrated electrons (*e_aq_*^−^), which played a key role in PFAS degradation by initiating the stepwise degradation pathway (Equations (11)–(14)). Over the 30-day period, a slight decrease in membrane permeability was observed which was attributed to the fouling of the membrane; as such, increasing crossflow velocity and modifying cleaning protocols to maintain performance were suggested for long-term operation.

(11) $$C_{7}F_{15}{COO}^{-}+e_{aq}^{-}\to {C_{7}F_{15}}^{∙}+{CO}_{2}$$

(12) $${C_{7}F_{15}}^{∙}+H_{2}O\to C_{7}F_{15}OH$$

(13) $$C_{7}F_{15}OH\to C_{6}F_{13}COF+HF$$

(14) $$C_{6}F_{13}COF\to {C_{6}F_{13}}^{∙}HF$$

### 4.2. Photocatalytic Membrane Reactors with Immobilized Photocatalyst

**Figure 19:**
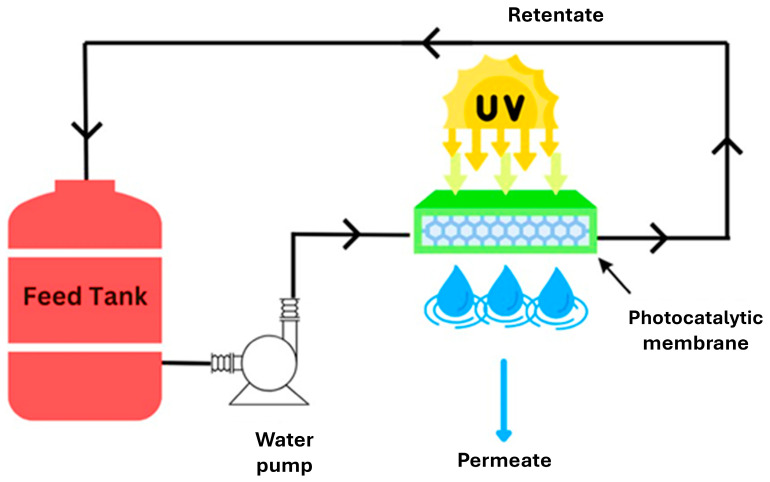
Schematic representation of an immobilized photocatalytic membrane reactor with crossflow filtration system.

In a system utilizing photocatalytic membranes (PMs), the membrane performs a dual function: acting as a support for the photocatalysts and serving as a selective barrier for pollutants keeping them within the reaction environment for degradation [105]. Reactors with immobilized photocatalysts are considered integrative-type photocatalytic membrane reactors (PMRs) combining both membrane separation and photocatalytic processes, as depicted in Figure 19 [102]. 

PMRs generally outperform conventional membranes by exhibiting improved permeate quality as not only do they reject PFASs, but they also degrade them, leading to a higher removal efficiency and reduced membrane fouling. Photocatalytic membranes achieve this by degrading organic pollutants in feed solutions through the generation of oxygen-reactive radicals under irradiation with light. This process prevents a cake layer from being formed on the membrane surface, reduces the concentration of pollutants in the retentate and enhances the quality of the permeate. For practical applications, reactors with immobilized photocatalysts are preferred due to their ability to operate continuously, eliminating the need for catalyst particle separation and enabling the reuse of catalytic supports for multiple cycles. However, immobilized systems may face challenges such as a lower area-to-volume ratio, which can lead to mass transfer limitations and reduced reaction rates which can be mitigated by implementing turbulent flow conditions [105].

Eke et al. [106] developed a hybrid phosphorene membrane and subjected it to UV irradiation for the degradation of PFOA. Initially, the PFOA-contaminated solution (100 mg/L) was passed through the membrane using crossflow filtration where PFOA was adsorbed onto the membrane. After filtration, the membrane underwent UV irradiation and oxygenation. Membranes exposed to UV (365 nm) for 120 min exhibited a removal rate of 91.95% of the adsorbed PFOA, while the membranes exposed to UV for 200 min achieved a removal rate of 98.4%. This enhancement was attributed to the photocatalytic properties of the membrane due to phosphorene; thus, PFOA was fragmented into smaller compounds during treatment. Post-treatment, the UV-treated membranes exhibited smoothness, hydrophilicity and minimal fluorine residue on the surface.

**Figure 20:**
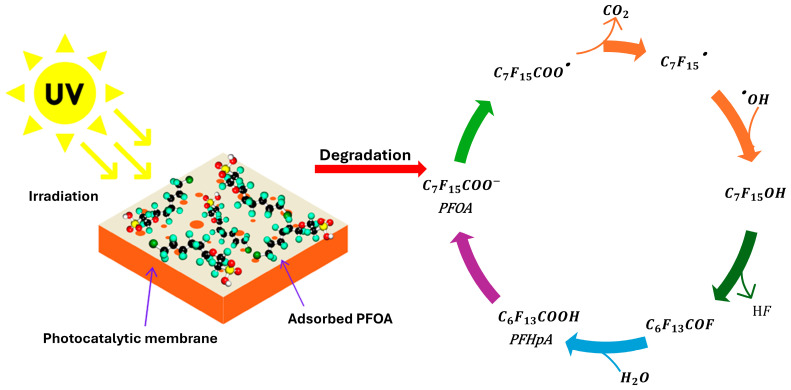
A schematic diagram showing the photocatalytic degradation of PFOA adsorbed onto the photocatalytic membrane after exposure to UV.

In the same vein, Junker et al. [107] employed UF membranes and used adsorptive Fe/TNT@AC as the photocatalyst. The photocatalyst was deposited onto the membrane via vacuum filtration after grafting with polydopamine (PDA) or plasma-activated acrylic acid (AA). The PFOA feed solution (10 ppb) was first circulated through the dead-end filtration system without UV irradiation to maximize interaction between the coated membranes and the PFAS solution, promoting the adsorption of PFASs onto the membrane surface. After a set period, the membranes were exposed to UV irradiation, triggering the degradation of the adsorbed PFASs. This degradation process was initiated by direct hole oxidation, leading to the radicalization of PFAS molecules, as illustrated in Figure 20.

The coated UF membranes were found to remove 80% of PFOA during the first two hours of the first cycle. However, after 8 h of continuous operation, the removal rate significantly decreased to 41%. In the second cycle, although the initial removal rate was slightly lower at 69%, the performance remained stable throughout the 8 h period. A mixed PFAS solution containing PFOA, PFOS, PFBA and PFBS was then used, and the removal of PFOA slightly decreased to 68% over an 8 hr cycle, while 79% of PFOS was removed, which then drastically diminished to 17% and 23% in the second cycle due to the saturation of active sites which are not quickly regenerated for more uptake. 

The significant decline in removal efficiency is, however, a drawback, requiring further optimization focusing on the stability of membranes making it suitable for real-world applications where continuous operation is required. A mass balance analysis of the used photocatalytic membranes revealed that 1% of PFBA, 2% of PFOA, 4% of PFBS and 44% of PFOS were adsorbed onto the membrane. This suggested that PFOS was primarily removed through adsorption, while PFOA was mainly degraded.

### 4.3. Membrane Fouling

Fouling in PMRs is commonly associated with slurry reactors, where the accumulation of photocatalyst nanoparticles on the membrane surface accelerates fouling, leading to a reduction in permeate flux [102]. The fouling of the membrane has been found to reduce permeate flux and productivity while increasing the time needed to clean the membrane. This reduces the lifespan of the membrane, resulting in an increase in costs [99]. Immobilized photocatalytic membrane reactors (PMRs) have demonstrated lower fouling rates than conventional membranes because the incorporated photocatalyst restricts the adsorption of particles onto the membrane surface. In slurry reactors, choosing an appropriate operating mode is crucial to mitigate membrane fouling. The crossflow mode is ideal for industrial applications, owing to its effectiveness in limiting fouling. In this mode, water flows parallel across the membrane surface which helps remove particles which are deposited on the surface which in turn reduces fouling [102,105]. The filtrate moves perpendicularly through the membrane, whilst the retentate is typically recirculated back to the feed tank, ensuring continuous operation and minimal fouling impact.

Various factors, including feedwater composition, photocatalyst loading, solute concentration and pressure, play a substantial role in the performance and development of a cake layer on the membrane surface. Studies indicate that increasing TMP initially enhances permeate flux until reaching an optimal pressure, beyond which further increases lead to cake formation [100]. This is because higher TMPs result in increased flow rates, leading to inevitable membrane fouling. Although high flow rates can also increase permeate flux, they contribute to fouling due to concentration polarization, especially at elevated flow rates, causing membrane fouling. For instance, PFAS molecules are rejected by the membrane at high flow rates, resulting in the formation of a dense cake layer and increased membrane resistance against permeability. With regard to photocatalyst loading, increasing it can reduce membrane foulants by enhancing the membrane hydrophilicity and accelerate photocatalytic degradation due to the increased reaction surface area. However, there is an optimal value for photocatalyst loading in each specific process, beyond which the enhanced murkiness of the reaction mixture prevents the absorption of light by the photocatalyst and the possibility of leaching [99].

## 5. Challenges and Recommendations

### 5.1. Limitations to Large-Scale Applications

Although the efficient removal of PFASs has been studied using photocatalysis, membrane separation and photocatalytic membrane systems, their application to long-term operation at full scale presents a lot of challenges; many studies rely on idealized models that do not account for real-world complexities, leading to discrepancies between predicted and actual performance metrics. A few things to consider when scaling up include the following:

- Light irradiation: The efficiency of photocatalysis often depends on light penetration depth. In large-scale systems, uneven light distribution can lead to reduced effectiveness.
- Reaction kinetics: The kinetics of PFAS degradation can be influenced by numerous factors, including the presence of competing substances, pH levels and temperature. These factors can hinder photocatalytic activity and lead to lower observed degradation efficiencies than those reported in theoretical studies.
- Real water matrices: The presence of competing substances in real wastewater can inhibit photocatalytic reactions, affecting the overall efficiency and selectivity of PFAS degradation.
- Fouling: Membrane fouling is a significant challenge that can reduce efficiency and increase operational costs. In large-scale systems, managing fouling is complex and requires effective cleaning protocols.
- Material compatibility: The materials used for membranes and photocatalysts must be compatible to avoid degradation, which can complicate the design and increase costs.

### 5.2. Recommendations

- To overcome the challenge of light distribution in full-scale operation, innovative reactor designs must be developed to ensure sufficient light distribution to photocatalysts, thereby facilitating effective photocatalysis.
- The development of support materials such as membranes for photocatalysts should be prioritized to ensure adequate interaction between the photocatalyst and PFASs to enhance degradation.
- Implementing systems with built-in cleaning systems for membrane separation can help mitigate fouling and prolong the lifespan of membranes.
- More pilot-scale studies should be conducted to assess the effectiveness of new nanomaterials under real-world conditions.
- More cost analyses should be conducted, and areas where costs can be reduced should be identified and implemented.
- The mass balance of fluorine should be carried out as part of the destructive technologies of PFAS.

## 6. Conclusions

The remediation of PFASs using photocatalysis, membrane separation and photocatalytic membrane reactors, emphasizing the importance of understanding factors that influence the performance of these methods, was explored in this paper. The factors highlighted how PFAS molecules interact with the materials used as photocatalysts and for membrane fabrication, which highlights the necessity of selecting materials specifically effective against PFASs. This research paves the way for developing advanced materials and methods to enhance PFAS removal. Despite existing studies on these materials’ effectiveness in removing PFASs from water, there is a notable lack of data on their real-world efficacy, particularly due to various water interferences. Additionally, most studies have focused on anionic PFASs such as PFOA and PFOS, leaving a gap in understanding the performance of these materials against other PFAS classes, including cationic and zwitterionic types. Future research should address these gaps by testing materials with a broader range of PFASs and developing new membrane technologies that improve removal efficiency, operate effectively at lower pressures, reduce fouling and can be used at full scale.

## Figures and Tables

**Table 1:** Performance of metal oxides in degradation of PFOA.

Photocatalyst	Type of PFAS	Experimental Conditions	Light Source	Degradation Efficiency (%)	Time of Reaction (min)	Reference
In_2_O_3_ microspheres	PFOA	[PFOA] = 30 mg/L pH = 3.9	UV (254 nm)	100	20	[42]
In_2_O_3_ nanocubes	PFOA	[PFOA] = 30 mg/L pH = 3.9	UV (254 nm)	100	40	[42]
In_2_O_3_ nanoplates	PFOA	[PFOA] = 30 mg/L pH = 3.9	UV (254 nm)	100	120	[42]
P25 TiO_2_	PFOA	[PFOA] = 30 mg/L pH = 3.9	UV (254 nm)	28.5	180	[42]
Needle-like Ga_2_O_3_	PFOA	[PFOA] = 500 μg/L [Ga_2_O_3_] = 0.5 g/L Temp = 25 °C pH = 4.7	UV (254 nm) VUV (185 nm)	100 100	60 40	[26]
Sheaf-like Ga_2_O_3_	PFOA	[PFOA] = 500 μg/L [Ga_2_O_3_] = 0.5 g/L Temp = 25 °C pH = 4.7	UV (254 nm)	100	45	[43]
CeO_2_	PFOA	[PFOA] = 50 mg/L [CeO_2_] = 0.5 g/L pH = 3.0	UV (254 nm)	40	600	[44]
ZnO	PFOA	[PFOA] = 53 mg/L [ZnO] = 0.53 g/L Temp = 30 °C pH = 6.5–7.0	Visible light (400–800 nm)	64	360	[45]

**Table 2:** Performance of modified TiO_2_ in PFOA degradation.

Photocatalyst	Type of PFAS	Operational Parameters	Light Source	Degradation Efficiency (%)	Defluorination Ratio	Time of Reaction (h)	Reference
TiO_2_	PFOA	[PFOA] = 60 mg/L pH = 3.0 [catalyst] = 0.5 g/L	UV (365 nm)	31.1	3.1	7	[49]
Ag-TiO_2_				57.7	8.1	7	[4]
Pd-TiO_2_				94.2	25.9	7	[49]
Pt-TiO_2_				100	34.8	5	[49]
Fe-TiO_2_	PFOA	[PFOA] = 50 mg/L pH = 5.0 [catalyst] = 0.5 g/L	UV (254 nm)	69	9	12	[34]
Cu-TiO_2_	PFOA		UV (254 nm)	91	19	12	[34]
Pb-TiO_2_	PFOA	[PFOA] = 50 mg/L pH = 5.0 [Pb-TiO_2_] = 0.5 g/L	UV (254 nm)	99.9	22.4	12	[39]
TiO_2_-rGO	PFOA	[PFOA] = 99.4 mg/L pH = 3.8 [TiO_2_-rGO] = 0.1 g/L	UV (254 nm)	93	-	12	[50]
TiO_2_-Pb/rGO	PFOA	PFOA] = 10 mg/L pH = 4.5–7.0 [TiO_2_-Pb/rGO] = 0.33 g/L	UV (254 nm)	98	32	24	[50]
TiO_2_-MWCNT	PFOA	[PFOA] = 30 mg/L pH = 5.0 [TiO_2_–MWCNT] = 1.6 g/L	UV (365 nm)	~100	-	8	[51]
TiO_2_-GO	PFOA	[PFOA] = 5 mg/L pH = 4.64 [TiO_2_-GO] = 200 mg/L	UV (254 nm)	93.6	-	8	[40]

## Data Availability

No new data were created or analyzed in this study. Data sharing is not applicable to this article.
